# Hybrid Plasma Spray Synthesis of Spherical Si_0.8_Ge_0.2_ Alloy Nanoparticles for Lithium-Ion Battery Anodes

**DOI:** 10.3390/nano15221718

**Published:** 2025-11-13

**Authors:** Wen-Bo Wang, Wenfang Li, Jun Du, Ryoshi Ohta, Makoto Kambara

**Affiliations:** 1 School of Materials Science and Engineering, Dongguan University of Technology, Dongguan 523808, China; w-b.wang@hotmail.com; 2 School of Materials Science and Engineering, South China University of Technology, Guangzhou 510640, China; jundu@scut.edu.cn; 3 Department of Materials Engineering, The University of Tokyo, 7-3-1, Hongo, Bunkyo, Tokyo 113-8656, Japan; ohta_r@plasma.t.u-tokyo.ac.jp; 4 Department of Materials and Manufacturing Science, Osaka University, 2-1, Yamadaoka, Suita, Osaka 565-0871, Japan; mkambara@mapse.eng.osaka-u.ac.jp

**Keywords:** plasma spraying, silicon-germanium alloy, nanoparticles, lithium-ion batteries, anode materials, co-condensation

## Abstract

Despite its ultrahigh theoretical capacity, silicon anodes for lithium-ion batteries suffer from severe capacity decay caused by over 300% volume changes during cycling. While Si–Ge alloying and spherical nanostructuring have been demonstrated to improve ionic/electronic transport and mechanical resilience, scalable synthesis of homogeneous, sub-150 nm SiGe nanospheres from low-cost precursors remains challenging. Here, we report a hybrid plasma-spraying physical vapor deposition (PS-PVD) process that directly converts metallurgical-grade Si and Ge powders into phase-pure Si_0.8_Ge_0.2_ nanospheres (<100 nm) at a continuous rate of 1 g min^−1^. The co-condensation mechanism during formation was elucidated through molecular dynamics (MD) simulations, which revealed a process initiated by inhomogeneous nucleation and followed by uniform cluster growth and spheroidization. Multiscale characterization confirmed the spherical morphology, compositional uniformity, and crystalline structure of the produced Si_0.8_Ge_0.2_ nanoparticles. The resulting anodes exhibited a stable capacity of ~1500 mAh g^−1^ at 0.1C over 100 cycles (>80% retention) and a Coulombic efficiency of ~98%. This approach bridges the gap between high-performance design and industrial manufacturability, offering a practical route to next-generation anodes for electric vehicles.

## 1. Introduction

Silicon (Si) is a promising anode material for next-generation lithium-ion batteries (LIBs), owing to its ultrahigh theoretical capacity (∼3579 mAh g^−1^ for Li_15_Si_4_), a low operating potential (~0.4 V vs. Li/Li^+^), and natural abundance [1]. Nevertheless, its practical application is hindered by a substantial volume expansion (∼300–400%) during (de)lithiation, which induces severe mechanical fracture, electrical contact loss, and continuous consumption of electrolytes [2]. These issues collectively manifest as rapid capacity fading and poor Coulombic efficiency (CE). Conventional approaches to mitigate these problems, such as nanostructuring (e.g., nanowires [3,4,5,6] or nanosheets [7,8,9]), compositing (e.g., Si/C core–shell architectures [10,11,12]), and alloying [13], alleviate volumetric strain but often face scalability challenges in laboratory syntheses. Conversely, industrially viable methods like wet chemistry or ball milling frequently result in particle aggregation and microcrack formation, undermining the intended mechanical benefits [14].

From a mechanical perspective, constructing spherical morphologies presents an effective solution [15]. The sphere represents the ideal shape to uniformly distribute stress and inhibit crack propagation [16]. Consistent with this principle, nanomechanical simulations by Yurkiv et al. [17] confirm that spherical Si nanoparticles mitigate cross-sectional expansion, reducing stress by 20–30%. McDowell et al. [18] further highlight the efficacy of spherical nanoparticles in suppressing radial stress concentrations, thereby promoting cyclability.

To realize such spherical architectures efficiently, plasma spraying physical vapor deposition (PS-PVD) has emerged as a robust technique [19]. This method offers potential for high throughput (up to kg h^−1^ scales [20,21]). Based on plasma fluid dynamics simulations [22], the quenching rate is estimated to be on the order of 10^6^–10^7^ K/s, a rate sufficient to enable a transient liquid-like state during co-condensation, allowing surface tension to drive spheroidization before solidification. Moreover, the PS-PVD process is uniquely suited for in-flight alloying via co-condensation in plasma. Alloying with germanium (Ge) is particularly attractive, as Ge possesses superior electronic conductivity and Li^+^ diffusivity (∼400 times faster than Si) [23]. Following Vegard’s law, Si–Ge forms solid solutions across compositions, which optimizes phase transitions during cycling. For instance, Duveau et al. [24] reported a capacity of ~1020 mAh g^−1^ after 100 cycles for a Si_0.9_Ge_0.1_ alloy, while Stokes et al. [25] achieved capacities of up to 1360 mAh g^−1^ with Si_0.67_Ge_0.33_ nanowires after 250 cycles.

Based on these design principles, we report a scalable synthesis of homogeneous Si_0.8_Ge_0.2_ nanospheres (<100 nm, throughput 1 g min^−1^) via a hybrid plasma spraying PVD process from low-cost raw metallurgical-grade Si (mg-Si) powders. Molecular dynamics simulations were employed to elucidate the co-condensation dynamics, revealing how inhomogeneous nucleation yields uniform cluster precursors that facilitate sphericity during growth for systems with different Ge contents. Multi-scale characterizations confirm the spherical morphology, compositional homogeneity, and structural integrity of the synthesized nanoparticles. Electrochemically, this material exhibits superior performance, including a high specific capacity of 1500 mAh g^−1^ at 0.1C over 100 cycles and a stable Coulombic efficiency of ~98%. Our PS-PVD process thus delivers a rare combination: atomic-level compositional control, spherical morphology, and relatively high throughput—all from low-cost mg-Si feedstocks—providing a realistic pathway toward commercial Si-alloy anodes.

## 2. Methodology

Si_0.8_Ge_0.2_ nanoparticles (NPs) were synthesized using a direct current–radio frequency (DC-RF) hybrid plasma spraying physical vapor deposition (PS-PVD) system. Raw metallurgical-grade Si powder (~20 μm, purity 99.5%) and Ge powder (~5 μm, purity 99.9%) were pre-mixed at a 4:1 atomic ratio, ball-milled, and fed axially at 1 g min^−1^ into the high-temperature plasma core (>6000 K) for complete vaporization. The vapor mixture subsequently underwent co-condensation and rapid quenching (10^6^–10^7^ K/s) in a water-cooled quenching vessel, forming spherical NPs. The detailed setup, gas flow dynamics, and synthesis parameters (Table S1) are provided in the Supplementary Information S1.1.

The morphology, composition, and crystal structure of the synthesized nanoparticles were characterized using field-emission scanning electron microscopy (FE-SEM) with energy-dispersive X-ray spectrometry (EDS), high-resolution transmission electron microscopy (HRTEM), scanning transmission electron microscopy (STEM) modes with high-angle annular dark-field (HAADF) detection, and X-ray diffraction (XRD). Surface chemistry was analyzed by X-ray photoelectron spectroscopy (XPS). Specific instrument models and operational details are summarized in the Supplementary Information S1.2.

The working electrode was fabricated by coating a slurry of active material (Si_0.8_Ge_0.2_ NPs, 60 wt%), Super-P carbon black (15 wt%), and polyimide binder (25 wt%) onto a copper foil. Electrodes were dried and roll-pressed (10 kN) before assembling 2016-type coin cells in an Ar-filled glovebox, using lithium metal as the counter/reference electrode and 1 M LiPF_6_ in EC/DEC/FEC (1:1:0.2) as the electrolyte. Galvanostatic charge–discharge tests were conducted between 0.01 and 1.5 V vs. Li/Li^+^. Galvanostatic cycling was performed using an ACD-M01A tester (Asuka Co., Ltd., Tokushime, Japan) at 0.02C (initial 1–3 cycles) and 0.1C thereafter, within 0–1.5 V vs. Li/Li^+^ at 25 °C. Capacities were normalized to the active mass, and the Coulombic efficiency (CE) was calculated from the ratio of charge to discharge capacity. For detailed electrode preparation and electrochemical testing protocols, refer to Supplementary Information S1.3.

Molecular dynamics (MD) simulations were performed using SCIGRESS ME software (Fujitsu Co., Ltd., Kanagawa, Japan) to elucidate co-condensation, inhomogeneous nucleation, and sphericity dynamics. The simulations utilized the original Tersoff three-body potential [26] for Si–Si, Si–Ge, and Ge–Ge interactions, which has been widely applied in Si–Ge vapor-phase nucleation and alloying studies [27,28,29,30]. While this potential slightly underestimates the melting point of pure Si (~1600 K vs. experimental 1687 K) [31,32,33], it reliably captures relative trends in nucleation sequence, compositional homogeneity, and surface-energy-driven spheroidization under rapid quenching conditions. Ar–Si/Ge interactions were modeled using Lennard-Jones potentials with Lorentz–Berthelot mixing rules. The simulations were conducted on systems containing 1000 Si/Ge atoms with varying compositions (Si_0.8_Ge_0.2_, Si_0.5_Ge_0.5_, Si_0.2_Ge_0.8_) in 15 nm cubic cells. To mimic the experimental quenching process, the systems were rapidly cooled from 6000 K to 1000 K at 5 × 10^12^ K/s. It should be noted that this cooling rate is significantly higher than that in experimental conditions due to computational limitations inherent to MD timescales. Nevertheless, the simulation captures the fundamental thermodynamic and kinetic trends, such as the sequence of nucleation and the driving force for spheroidization. Clusters were identified via Stillinger criterion (cutoff 2 times bond length) [34]. Analysis was performed using OVITO for composition and distribution [35]. Detailed protocols are provided in the Supplementary Information S1.4.

## 3. Results and Discussion

### 3.1. Synthesis Mechanism and Structural Evolution of Si-Ge Nanospheres

**Figure 1:**
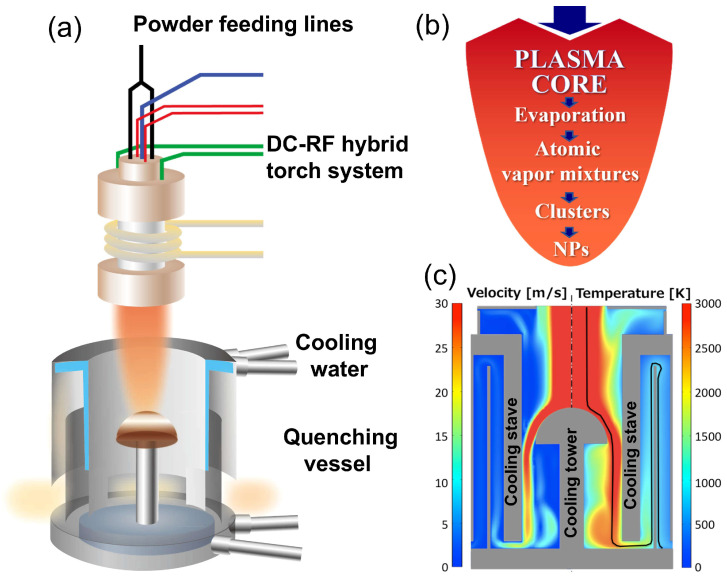
Schematic illustration of the PS-PVD for spherical nanoparticle synthesis via condensation utilizing a DC-RF hybrid plasma system. (**a**) Powder feeding lines integrated with a DC-RF hybrid torch system, showing the injection of raw powders into the high-temperature plasma jet and water-cooled quenching vessel, are placed in the chamber for powder collection. (**b**) Conceptual representation of the plasma core, highlighting sequential processes of evaporation, atomic vapor mixtures, cluster formation, and spherical nanoparticle (NP) growth. (**c**) Simulated axial profiles of gas velocity (m/s, **left** scale) and temperature (K, **right** scale) within the water-cooled quenching vessel equipped with a hemispherical copper cooling tower, under the conditions detailed in Supplementary Table S1, highlighting staged quenching from the cooling tower (gas quenching, high T) to the cooling stave (wall quenching, low T) that promotes formation of spherical NPs.

Figure 1 schematically illustrates the DC-RF hybrid plasma spraying physical vapor deposition (PS-PVD) system for synthesizing spherical Si-Ge nanoparticles via co-condensation. In Figure 1a, the setup features a hybrid plasma torch that synergistically combines inductively coupled plasma (ICP, generated by RF power at 90 kW) for a large-volume high-temperature zone and a direct current (DC) jet (8 kW) for flow stabilization and axial powder injection. This design allows precursor powders to be fed directly into the plasma core without disturbance from recirculation eddies, ensuring complete vaporization at temperatures exceeding 6000 K and enabling high production throughput from low-cost feedstock.

The physicochemical transformations are conceptually outlined in Figure 1b. Upon injection, Si and Ge powders evaporate instantaneously into atomic vapor mixtures. As the plasma flux enters the quenching vessel, rapid cooling induces supersaturation, triggering inhomogeneous nucleation and co-condensation of Si-Ge clusters. These clusters grow by vapor accretion and coalescence. When they exist in a liquid-like state, the high surface tension encourages spheroidization, and they eventually solidify into homogeneous spherical nanoparticles. Unlike classical vapor–liquid–solid (VLS) growth that relies on catalytic seeds [36], our plasma-driven condensation proceeds via transient liquid-like clusters that rapidly solidify—enabling homogeneous alloying without catalysts or post-annealing, as evidenced by Vegardian lattice expansion and uniform Ge distribution.

Figure 1c depicts simulated axial profiles of gas velocity (m/s, left scale) and temperature (K, right scale) within the water-cooled quenching vessel equipped with a hemispherical copper cooling tower, computed using COMSOL 6.0 Multiphysics under experimental conditions (Supplementary Table S1). While direct in situ validation was not feasible under the extreme plasma conditions, the model is consistent with established literature data [19,20,21,22] and successfully reproduces the observed nanoparticle morphology and production rate, supporting its qualitative reliability for process interpretation. The profiles show a staged quenching mechanism: the high-temperature plasma gas (>6000 K at inlet) cools down to 2000–3000 K in the free jet region, which controls nucleation and early cluster growth below the Si homogeneous nucleation temperature (~2200 K). After hitting the walls of the chamber, the cooling process speeds up to less than 1000 K, which helps with heat extraction and controls solidification. This staged quenching—initial gas-phase cooling followed by wall-contact heat transfer—facilitates controlled co-condensation, bypassing prolonged annealing and enabling spherical NP formation at rates compatible with industrial scalability. Final products were collected after cooling to room temperature, yielding NPs with diameters < 100 nm.

**Figure 2:**
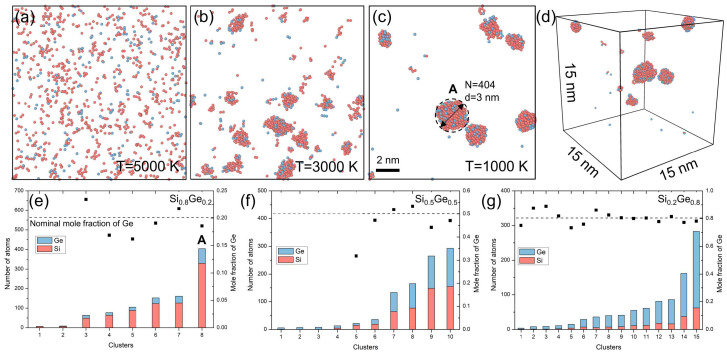
Molecular dynamics (MD) simulations of inhomogeneous nucleation and cluster growth in the Si-Ge (Si (red) and Ge (blue)) binary vapor system during plasma co-condensation. Atomic configuration of Si_0.8_Ge_0.2_ case at (**a**) T = 5000 K, showing initial nucleation. (**b**) Cluster formation at T = 3000 K. (**c**) Coalesced clusters at T = 1000 K, exhibiting amorphous transient spherical NPs (inset: the largest cluster A with N = 404 atoms and d ≈ 3 nm). (**d**) Three-dimensional rendering of Si-Ge mixed cluster in the cell of 15 nm scale for the Si_0.8_Ge_0.2_ case at T = 1000 K. Number and mole fraction of atoms in clusters at T = 1000 K for (**e**) the Si_0.8_Ge_0.2_ case, (**f**) the Si_0.5_Ge_0.5_ case, and (**g**) the Si_0.2_Ge_0.8_ case, revealing size-dependent compositional inhomogeneity.

Due to the high temperature, high speed, and complexity of the plasma process illustrated in Figure 1, it is challenging to observe the co-condensation mechanism directly at the atomic level. Therefore, we employed molecular dynamics (MD) simulations to investigate the nucleation and cluster growth in the Si–Ge binary vapor system. Figure 2 illustrates the MD simulation results for the Si-Ge system during plasma co-condensation, with Si atoms in red and Ge atoms in blue. The snapshots (a) at T = 5000 K, (b) at T = 3000 K, and (c) at T = 1000 K show the evolution from initial vapor mixing to cluster formation and growth for the present Si_0.8_Ge_0.2_ case. At 5000 K, the system remains in a well-mixed gaseous state. As the temperature decreases to 3000 K, small clusters begin to nucleate, exhibiting irregular and loosely bonded structures due to high atomic mobility and incomplete coalescence. Upon further cooling to 1000 K, the clusters grow larger and adopt more spherical morphologies, driven by surface energy minimization. The largest cluster at this stage contains *N* = 404 atoms with a diameter of approximately 3 nm (Figure 2c, inset). The three-dimensional rendering in Figure 2d highlights the overall sphericity of the resulting nanoclusters within the 15 nm simulation cell.

To further evaluate the compositional evolution, Figure 2e–g present the cluster size and Ge mole fraction for three system compositions (Si_0.8_Ge_0.2_, Si_0.5_Ge_0.5_, and Si_0.2_Ge_0.8_) at 1000 K. In all cases, the Ge fraction in larger clusters converges toward the nominal composition, though some size-dependent fluctuations are observed—particularly in Si-rich systems (Figure 2e), where the Ge fraction oscillates near the expected value of 0.2. This indicates that, despite initial compositional variations during nucleation, the final clusters are well-mixed and reflect the global stoichiometry, independent of cluster size. The simulation results reveal an inhomogeneous nucleation mechanism wherein Si-rich nuclei form preferentially due to their lower nucleation barrier, followed by incorporation of Ge atoms and structural spheroidization during coalescence [37,38]. This atomic-scale pathway corroborates the experimental observation of homogeneous Si_0.8_Ge_0.2_ nanospheres, confirming that rapid plasma quenching promotes initial Si-rich nucleation but ultimately leads to uniformly mixed, spherical nanoparticles via co-condensation. These findings confirm the significance of the PS-PVD process in attaining the morphological and compositional uniformity necessary for high-performance Si–Ge anode materials.

### 3.2. Morphological and Compositional Characterization of Si_0.8_Ge_0.2_ Nanospheres

**Figure 3:**
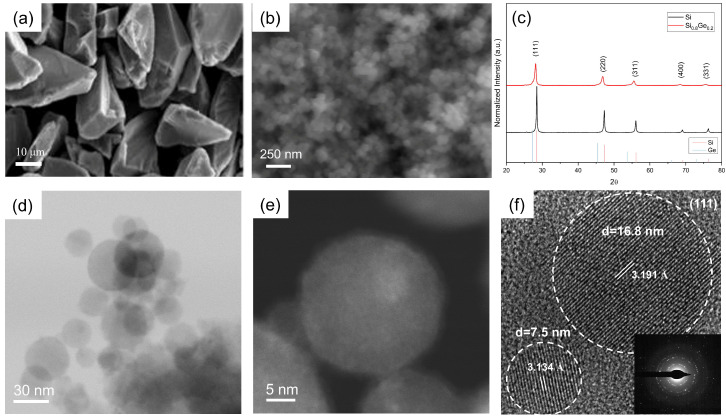
Multi-scale morphological and structural characterization of plasma-synthesized Si_0.8_Ge_0.2_ nanoparticles. SEM image of (**a**) the raw mg-Si powders and (**b**) plasma-sprayed Si_0.8_Ge_0.2_ NPs. (**c**) Normalized XRD spectrum of Si_0.8_Ge_0.2_ and as-prepared pure Si NPs. (**d**) STEM bright-field image, (**e**) HAADF-STEM image, and (**f**) HRTEM of produced Si_0.8_Ge_0.2_ nanoparticles, highlighting perfect spherical structures. The HRTEM lattice fringe analysis and the corresponding SAED pattern (inset) of the nanoparticles indicate a particle size of approximately d = 16.8 nm, with interplanar spacings of 3.191 Å and d = 7.5 nm (3.134 Å) for both (111) planes.

Figure 3 presents a multi-scale morphological and structural characterization of the plasma-synthesized Si_0.8_Ge_0.2_ nanoparticles. The SEM image in Figure 3a shows the raw micron-sized Si powders with a crushed morphology and a mean diameter of approximately 20 μm. In contrast, the plasma-sprayed Si_0.8_Ge_0.2_ nanoparticles in Figure 3b are agglomerated but exhibit a globular shape with sizes below 100 nm. This transformation from large, irregular raw particles to nanosized spheres is indicative of complete vaporization of the feedstock followed by rapid co-condensation in the plasma process, which promotes uniform nucleation and growth. The normalized XRD spectrum in Figure 3c compares the Si_0.8_Ge_0.2_ nanoparticles with as-prepared pure Si nanoparticles. All diffraction peaks correspond to crystalline structures, confirming the formation of alloyed phases. A shift in the three main peaks toward lower angles compared to pure Si suggests lattice expansion, consistent with Vegard’s law for Si_1-x_Ge_x_ solid solutions [39]. This implies homogeneous alloying rather than a physical mixture of unreacted precursors, as the larger Ge atoms (covalent radius ≈ 122 pm) substitute for Si (≈111 pm), expanding the lattice parameter. Rietveld refinement (Supplementary Figure S1) yielded a lattice parameter of a = 5.481(2) Å, corresponding to a Ge content of 22.2% based on Vegard’s law, which is in good agreement with the nominal composition of Si_0.8_Ge_0.2_.

The STEM bright-field image in Figure 3d and HAADF-STEM image in Figure 3e further reveal the perfect spherical morphology of the Si_0.8_Ge_0.2_ nanoparticles, with diameters around 10–50 nm. The Z-contrast in HAADF-STEM (proportional to Z^2^) highlights the uniform distribution of heavier Ge atoms (Z = 32) within the lighter Si matrix (Z = 14), without significant segregation or phase separation at this scale. High-resolution TEM (HRTEM) in Figure 3f provides atomic-level insights, showing lattice fringes with interplanar spacings of 3.191 Å for a ~16.8 nm particle and 3.134 Å for a ~7.5 nm particle, both corresponding to the Si-Ge (111) planes (adjusted from pure Si’s 3.135 Å due to alloying). The inset SAED pattern confirms the crystalline nature, with rings indicating polycrystallinity in the ensemble but single-crystal-like order within individual particles.

**Figure 4:**
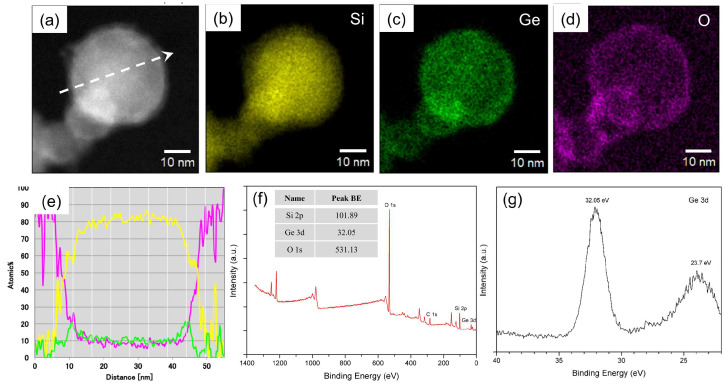
Elemental distribution and spectroscopic characterization of plasma-synthesized Si_0.8_Ge_0.2_ nanoparticles. (**a**) HAADF-STEM image of a single nanoparticle, with maps for (**b**) Si, (**c**) Ge, and (**d**) O, revealing uniform elemental distribution with surface oxidation. (**e**) Atomic intensity profile along a 50 nm line scan, demonstrating 80% Si content with oxygen enrichment at the surface. (**f**) XPS spectrum with peak binding energies (BE) for Si 2p (101.89 eV), Ge 3d (32.05 eV), and O 1s (531.13 eV). (**g**) High-resolution XPS spectrum of the Ge 3d region, with a peak at 32.05 eV and a shoulder at 23.7 eV, confirming Ge oxidation states in the nanoparticle matrix. Arrow in (**a**): line-scan direction.

Figure 4 presents the elemental distribution and spectroscopic characterization of the plasma-synthesized Si_0.8_Ge_0.2_ nanoparticles. Figure 4a shows an HAADF-STEM image of a single nanoparticle, revealing a globular morphology with a diameter of approximately 40 nm. The accompanying elemental maps for Si (b), Ge (c), and O (d) demonstrate a uniform distribution of Si and Ge throughout the particle core, indicating effective alloying during co-condensation in the plasma process. The O map reveals surface enrichment, suggesting that oxygen is primarily localized at the nanoparticle exterior rather than homogeneously dispersed within the matrix. This surface O layer, estimated to be a few nanometers thick based on the line scan profile in Figure 4e, likely originates from post-synthesis oxidation upon exposure to ambient air during particle collection, despite efforts to minimize it (e.g., via Ar purging). The profile confirms an average composition of approximately 80% Si and 10–20% Ge in the core, with O signals peaking at the surface boundaries. This ratio was quantified by EDS line-scan and mapping analyses focused on the interior region of individual nanoparticles, and is further corroborated by the Vegardian lattice expansion observed in XRD in Figure 3c, which corresponds to a Ge content of ~18–22 at.%. It underscores the core–shell-like structure, where the Si-Ge alloy forms the robust inner framework to accommodate volume expansion during lithiation/delithiation, while the surface O enrichment (up to ~10% locally) represents a passivation layer that, while consuming active material, may also modulate the SEI formation process [40].

XPS further probed the surface chemistry in Figure 4f,g. The survey spectrum in Figure 4f shows peaks for Si 2p, Ge 3d, and O 1s at binding energies indicative of oxidized species (Si 2p at 101.89 eV and Ge 3d at 32.05 eV). These values indicate substantial surface oxidation of both elements, as the Ge 3d peak is shifted from elemental Ge (~29 eV) to oxidized forms (~32–33 eV). The high-resolution XPS of the Ge 3d region in Figure 4g is dominated by a peak at 32.05 eV, indicative of GeO_x_ (e.g., GeO or GeO_2_). The origin of the minor shoulder at approximately 23.7 eV is unclear and may be attributed to an energy loss feature or other satellite peak, as its binding energy is inconsistent with that of metallic Ge^0^. This surface oxide layer, consistent with the EDS line scan in Figure 4e, is a common feature in nanomaterials and may lead to the initial irreversible capacity loss due to the irreversible consumption of lithium [41].

Overall, these structural features—nanosized spheres with homogeneous Si-Ge alloying and minimal defects—are crucial for battery performance. The small particle size (<150 nm) mitigates fracture during lithiation, as per critical size thresholds [42], while uniform composition enhances Li-ion diffusivity (~400 times faster in Ge than Si) and electronic conductivity, potentially improving rate capability and cyclability, as observed in similar alloyed systems [43,44].

### 3.3. Electrochemical Performance as LIB Anodes

**Figure 5:**
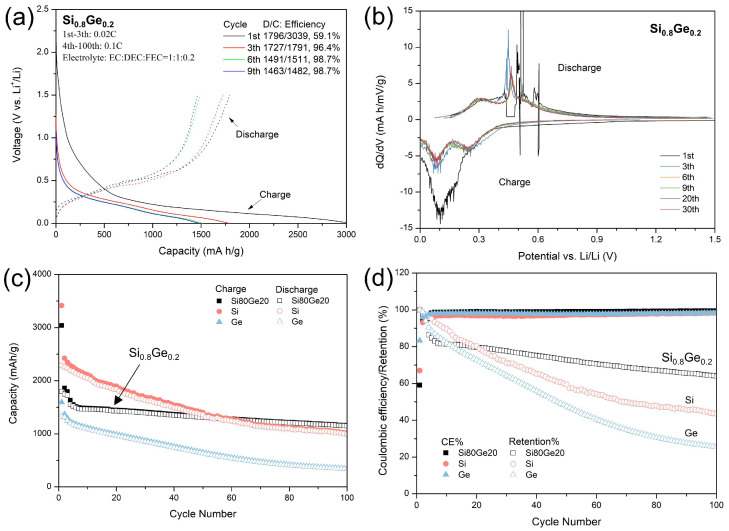
Electrochemical performance of half-cell lithium-ion batteries using plasma-synthesized Si_0.8_Ge_0.2_ nanoparticles as anodes. (**a**) Galvanostatic charge–discharge voltage profiles at initial several cycles (1st, 3rd, 6th, and 9th cycles), showing initial discharge/charge capacities and voltage plateaus indicative of Li-Si/Ge alloying. (**b**) Differential capacity (dQ/dV) plots versus potential (V vs. Li/Li^+^), highlighting redox peaks for lithiation/delithiation processes across cycles. (**c**) Specific capacity (mAh g^−1^) versus cycle number for Si_0.8_Ge_0.2_ (black). (**d**) Coulombic efficiency and capacity retention as a function of cycle number, illustrating enhanced stability with >98% efficiency after 100 cycles. As a benchmark, the corresponding performance profiles of as-prepared pure Si (red) and pure Ge (blue) anodes were added in (**c**,**d**).

The electrochemical performance of the plasma-synthesized Si_0.8_Ge_0.2_ nanospheres was evaluated in half-cell configuration, as shown in Figure 5. The galvanostatic charge–discharge voltage profiles for the initial cycles (Figure 5a) show sloping plateaus that are typical of Li alloying with Si-Ge. The first discharge capacity was 3039 mAh g^−1^, and the charge capacity was 1796 mAh g^−1^, yielding an initial Coulombic efficiency (ICE) of 59.1%, consistent with values reported for nanostructured Si-based systems [45,46]. The relatively low ICE is attributed to irreversible solid electrolyte interphase (SEI) formation and reduction in the native surface oxide layer (as evidenced in Figure 4), exacerbated by the high surface area of the nanospheres [47,48]. These profiles highlight the material’s high initial lithium uptake while revealing activation processes that stabilize subsequent cycling. Critically, the reversibility improves dramatically after the first cycle, with the average CE exceeding 98% over the subsequent cycles.

To probe the underlying reaction mechanisms, differential capacity (dQ/dV) plots are presented in Figure 5b. The broad peaks during lithiation (~0.1 V and 0.28 V) and the sharp delithiation peak at 0.45 V with a shoulder at 0.3 V evolve after the first cycle, reflecting the amorphization of crystalline Si_0.8_Ge_0.2_ during initial lithiation and the formation of a Li_15_(Si/Ge)_4_-like crystalline phase upon delithiation [49]. This phase transformation, well-documented in Si-Ge alloys, mitigates mechanical strain by accommodating volume changes in an amorphous matrix, contributing to enhanced cyclability. The long-term stability is demonstrated in Figure 5c,d, where the Si_0.8_Ge_0.2_ electrode is benchmarked against as-synthesized pure Si and Ge anodes. While pure Si/Ge exhibits rapid capacity decay due to pulverization, Si_0.8_Ge_0.2_ maintains ~1500 mAh g^−1^ after 100 cycles at 0.1C, with over 80% retention. This specific capacity is normalized to the mass of the active Si_0.8_Ge_0.2_ material only (60 wt% of the total electrode), excluding the polyimide binder (25 wt%) and Super-P carbon black (15 wt%), in accordance with standard practice for comparing intrinsic anode performance. The CE of both pure and alloyed plasma-synthesized NPs stabilizes above 98% after the initial 3 cycles, surpassing conventional nanostructured Si-based anodes (e.g., ball-milled variants [48]). A comprehensive comparison of our material’s performance metrics with previously reported SiGe-based anode systems is provided in Supplementary Table S2. This comparison highlights the unique combination of high-capacity retention and industrial-scale synthesis throughput (1 g min^−1^) achieved in our hybrid PS-PVD processed nanospheres.

The sustained capacity may arise from two interlinked factors: (i) Ge alloying accelerates Li^+^ transport (≈400 times faster than Si) and boosts electronic conductivity, mitigating polarization; and (ii) the sub-100 nm spherical morphology distributes lithiation-induced stress uniformly, suppressing crack propagation—as predicted by nanomechanical models [16,17]. Although initial oxide-related losses persist, prelithiation or coatings could further optimize practical viability [47]. This study confirms that the plasma-synthesized, homogeneous Si_0.8_Ge_0.2_ nanospheres represent a promising anode material, successfully balancing high specific capacity with long-term cycling stability.

## 4. Conclusions

In summary, our hybrid plasma spraying PVD process enables the continuous, one-step production of compositionally homogeneous Si_0.8_Ge_0.2_ nanospheres (<100 nm) directly from low-cost metallurgical-grade silicon—a combination rarely achieved in prior SiGe syntheses. The resulting material simultaneously leverages Ge’s fast Li^+^ diffusivity and the mechanical resilience of spherical morphology to suppress pulverization and stabilize the electrode/electrolyte interface. Consequently, the anode exhibits a reversible capacity of around 1500 mAh g^−1^ after 100 cycles at 0.1C, alongside a Coulombic efficiency of approximately 98%—performance that exceeds many conventional nanostructured Si/Ge anodes tested under comparable conditions.

The approach achieves a throughput of 1 g min^−1^ without the need for additional post-synthesis stages, which represents a significant advantage when transitioning nanomaterials from laboratory to industrial production. Although initial Coulombic inefficiency poses a limitation, strategies such as carbon coatings or prelithiation could effectively reduce irreversible lithium consumption. Overall, these findings position PS-PVD as a viable method for producing high-energy Si-alloy anodes that integrate readily with current battery fabrication processes.

## Data Availability

The datasets generated during the current study are available from the corresponding authors on reasonable request.

## Supplementary Materials

The following supporting information can be downloaded at: https://www.mdpi.com/article/10.3390/nano15221718/s1, Table S1: Experimental conditions of the hybrid PS-PVD process. Table S2: Comparison of electrochemical performance of Si-Ge alloy anodes. Figure S1: Rietveld refinement of the XRD pattern. The refined lattice parameter (a = 5.481(2) Å) corresponds to a Ge content of 22.2%, consistent with the target composition. References [50,51,52,53,54,55,56,57,58,59] are cited in the Supplementary Materials.

## Abbreviations

The following abbreviations are used in this manuscript:

FE-SEM	Field-emission scanning electron microscopy
EDS	Directory of open access journals
HRTEM	High-resolution transmission electron microscopy
STEM	Scanning transmission electron microscopy
HAADF	High-angle annular dark field
XRD	X-ray diffraction
XPS	X-ray photoelectron spectroscopy
NPs	Nanoparticles
PS-PVD	Plasma spraying physical vapor deposition
CE	Coulombic efficiency
MD	Molecular dynamics
DC-RF	Direct current–radio frequency
