# Supplementary material for: Hybrid Plasma Spray Synthesis of Spherical Si0.8Ge0.2 Alloy Nanoparticles for Lithium-Ion Battery Anodes
Source: Nanomaterials (Basel). 2025 Nov 13;15(22):1718. doi: 10.3390/nano15221718 (PMC12655563; doi:10.3390/nano15221718)
Supplement: Supplementary file 1 [file nanomaterials-15-01718-s001.zip › nanomaterials-3966406-supplementary.pdf]

Supplementary Information

Hybrid Plasma Spray Synthesis of Spherical $Si_{0.8}Ge_{0.2}$ Alloy Nanoparticles for Lithium-Ion Battery Anodes

Wen-bo Wang[a,b], Wenfang Li[a,*], Jun Du[b], Ryoshi Ohta[c], Makoto Kambara[c,d,]

[a] *Institute of Science&Technology Innovation, Dong Guan University of Technology, Dong Guan, 523000, China*

[b] *School of Materials Science and Engineering, South China University of Technology, Guangzhou 510640, China*

[c] *Department of Materials Engineering, The University of Tokyo, 7-3-1, Hongo, Bunkyo, Tokyo 113-8656, Japan*

[d] *Department of Materials and Manufacturing Science, Osaka University, 2-1, Yamadaoka, Suita, Osaka 565-0871, Japan*

* Corresponding author.

E-mail address: mewfli@163.com (W.F. Li);

S1. Methods

S1.1 PS-PVD Synthesis

The DC-RF hybrid plasma torch utilizes a 3-turn RF coil to generate inductively coupled plasma, stabilized by a superimposed DC jet. RF Ar (radial 140 slm, tangential 30 slm) and DC Ar (10 slm) form the primary plasma, with H_2 (50 slm) for added for enhanced vaporization efficiency. Pre-mixed Si/Ge powders were fed via TP-99010FDR feeder (JEOL Ltd.). The chamber pressure was maintained at 400 Torr. The powder feeding rate was 1 g/min. The quenching vessel features a hemispherical water-cooled copper tower to promote staged cooling, which facilitates the formation of uniform spherical nanoparticles. Computational fluid dynamics (CFD) simulations (Table S1 conditions) confirmed the temperature and flow fields conducive to NP formation. NPs detached under Ar purge to minimize oxidation.

Table S1. Experimental conditions of the hybrid PS-PVD process

Parameters	Value
DC power	8 kW
RF power	90 kW
Radial Ar	140 slm
Tangential Ar	30 slm
H_2 flow rate	50 slm
DC Ar	10 slm
Carrier Ar	3.6 slm
Feeding rate	1 g/min
Chamber pressure	400 Torr

S1.2 Materials Characterization

SEM/EDS (Hitachi H-800 FESEM) operated at 15 kV for imaging and 20 kV for EDS mapping. HRTEM/STEM (Hitachi High-Tech H-9500) operated at 200 kV with SAED. XRD (Bruker D2 PHASER) with Cu Kα radiation, 2θ range from 20° to 90°,

step size 0.02°. Surface chemistry and oxidation states were characterized by XPS (ESCALAB 250Xi, Thermo Fisher Scientific) with a monochromatic Al Kα source with binding energy calibration to C 1s at 284.8 eV.

S1.3 Electrode Preparation and Testing

The electrode slurry was prepared in N-methyl-2-pyrrolidone (NMP). The coated electrode was first pre-dried at 110 °C for 15 min under vacuum, then roll-pressed at 10 kN to a final thickness of ~20 μm, and finally dried again at 110 °C for 45 min under vacuum. The mass loading of the active material was 1–2 mg cm^{-2}. A Celgard 2400 polypropylene membrane was used as the separator. All electrochemical tests were performed in triplicate to ensure reproducibility.

S1.4 MD Simulation Protocol

All MD simulations were performed using SCIGRESS commercial software. Cluster analyses were applied within OVITO[1], which was also used for visualizations. The simulations were carried out in a cubic box with the cell length of 15 nm in NVT (constant number, volume, and temperature) ensemble. The periodic boundary conditions and the standard velocity-Verlet integrator were used, and the time-step was set to be 1 fs. The total target atoms number is 1000, and we focus on the $Si_{0.8}Ge_{0.2}$ systems with the 800 Si atoms and 200 Ge atoms. To mimic the real experiment closely, we added 1000 Ar atoms as a carrier gas to remove the latent heat in each run. To study the effect of different Ge contents on the cluster formation, we also added further runs by varying the Si/Ge ratio.

We heated the temperature of the system to 6000 K immediately and then cooled down the temperature to 1000 K within 1 ns. The original Tersoff 3-body potential[2,3] was used to describe the target interactions of Si-Si, Si-Ge, and Ge-Ge. Other interaction types (e.g., Ar-Ar, Ar-Si, Ar-Ge) were modeled by simply pair Lennard-Jones potential[4]. The unlike-pair interactions of Ar-Si and Ar-Ge were calculated by the Lorentz-Berthelot mixing rules[5]. The potential cutoff distance was set to be 15 Å for all the runs without tail corrections. Clusters were detected by the Stillinger criterion[6]. It

considers that two atoms belong to a cluster if the distance between them is smaller than a given value r_s = (1.5~2) σ, where σ is the bond length of the two atoms.

S2. Results

S2.1 Rietveld refinement

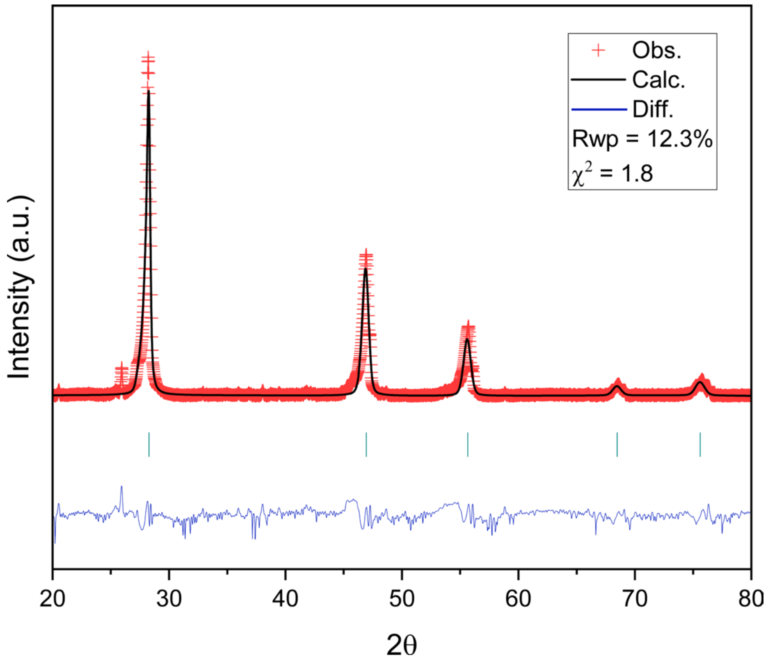

Fig. S1. Rietveld refinement of the XRD pattern. The refined lattice parameter (a = 5.481(2) Å) corresponds to a Ge content of 22.2%, consistent with the target composition.

S2.2 Electrochemical performance

Table S2. Comparison of electrochemical performance of Si-Ge alloy anodes.

**Material**	**Synthesis Method**	**Capacity (m Ah g^{-1})**	**Cycles**	**Current Density**	**CE (%)**
$Si_{0.8}Ge_{0.2}$ NPs (this work)	Hybrid PS-PVD	~1500	100	0.1C	~98
Sr-modified SiGe[7]	Dealloying	1166	100	0.1 A g^{-1}	83.62
Unmodified SiGe[7]	Dealloying	780	100	0.1 A g^{-1}	76.08

SiGe ($Al_{80}Si_{12}Ge_8$)[8]	Dealloying	1364	80	0.1 A g^{-1}	N/A
SiGe ($Al_{85}Si_9Ge_6$)[8]	Dealloying	1072	80	0.1 A g^{-1}	N/A
$Si_{0.67}Ge_{0.33}$[9]	N/A	1360	250	0.2C	N/A
Si/Ge hNWs[10]	Solution process	1040	150	0.2C	98.9
SiGe NWs[11]	N/A	>1031	400	0.2C	89.0
Nanoporous SiGe[12]	Liquid_Metal-Modified	~1200	300	0.2 A g^{-1}	N/A
Si1-xGex Films[13]	CVD	~1300	50	0.1C	N/A

Abbreviations: NPs = Nanoparticles; PS-PVD = Plasma Spray Physical Vapor Deposition; NWs = Nanowires; hNWs = heterostructure Nanowires; N/A = Not Available; CE = Coulombic Efficiency; CVD = Chemical Vapor Deposition.

References

(1) Stukowski, A. Visualization and Analysis of Atomistic Simulation Data with OVITO--the Open Visualization Tool. *Model Simul Mat Sci Eng* **2009**, *18* (1), 15012.

(2) Tersoff, J. Modeling Solid-State Chemistry: Interatomic Potentials for Multicomponent Systems. *Phys Rev B* **1989**, *39* (8), 5566–5568. https://doi.org/10.1103/PhysRevB.39.5566.

(3) Tersoff, J. Empirical Interatomic Potential for Silicon with Improved Elastic Properties. *Phys Rev B* **1988**, *38* (14), 9902.

(4) Wang, X.; Ramírez-Hinestrosa, S.; Dobnikar, J.; Frenkel, D. The Lennard-Jones Potential: When (Not) to Use It. *Physical Chemistry Chemical Physics* **2020**, *22* (19), 10624–10633. https://doi.org/10.1039/C9CP05445F.

(5) Delhommelle, J.; Millié, P. Inadequacy of the Lorentz-Berthelot Combining Rules for Accurate Predictions of Equilibrium Properties by Molecular Simulation. *Mol Phys* **2001**, *99* (8), 619–625. https://doi.org/10.1080/00268970010020041.

(6) Stillinger, F. H. Rigorous Basis of the Frenkel-Band Theory of Association Equilibrium. *J Chem Phys* **1963**, *38* (7), 1486–1494. https://doi.org/10.1063/1.1776907.

(7) Zhang, H.; Li, J. High-Performance SiGe Anode Materials Obtained by Dealloying a Sr-Modified Al–Si–Ge Eutectic Precursor. *RSC Adv* **2023**, *13* (4), 2672–2679. https://doi.org/10.1039/D2RA07674H.

(8) Yang, Y.; Liu, S.; Bian, X.; Feng, J.; An, Y.; Yuan, C. Morphology- and Porosity-Tunable Synthesis of 3D Nanoporous SiGe Alloy as a High-Performance Lithium-Ion Battery Anode. *ACS Nano* **2018**, *12* (3), 2900–2908. https://doi.org/10.1021/ACSNANO.8B00426.

(9) Stokes, K.; Geaney, H.; Flynn, G.; Sheehan, M.; Kennedy, T.; Ryan, K. M. Direct Synthesis of Alloyed Si1-XGex Nanowires for Performance-Tunable Lithium Ion Battery Anodes. *ACS Nano* **2017**, *11* (10), 10088–10096. https://doi.org/10.1021/ACSNANO.7B04523.

(10) Adegoke, T. E.; Abdul Ahad, S.; Bangert, U.; Geaney, H.; Ryan, K. M. Solution Processable Si/Ge Heterostructure NWs Enabling Anode Mass Reduction for Practical Full-Cell Li-Ion Batteries. *Nanoscale Adv* **2023**, *5* (23), 6514–6523. https://doi.org/10.1039/D3NA00648D.

(11) Kim, H.; Son, Y.; Park, C.; Lee, M. J.; Hong, M.; Kim, J.; Lee, M.; Cho, J.; Choi, H. C. Germanium Silicon Alloy Anode Material Capable of Tunable Overpotential by Nanoscale Si Segregation. *Nano Lett* **2015**, *15* (6), 4135–4142. https://doi.org/10.1021/ACS.NANOLETT.5B01257.

(12) Meng, F.; Wang, F.; Yu, H.; Zhao, Z.; Lv, Y.; Ma, C.; Zhang, D.; Liu, X. Liquid Metal-Modified Nanoporous SiGe Alloy as an Anode for Li-Ion Batteries and Its Self-Healing Performance. *ACS Appl Energy Mater* **2021**, *4* (12), 14575–14581. https://doi.org/10.1021/ACSAEM.1C03204.

(13) Abel, P. R.; Chockla, A. M.; Lin, Y. M.; Holmberg, V. C.; Harris, J. T.; Korgel, B. A.; Heller, A.; Mullins, C. B. Nanostructured Si(1-x)Gex for Tunable Thin Film Lithium-Ion Battery Anodes. *ACS Nano* **2013**, *7* (3), 2249–2257. https://doi.org/10.1021/NN3053632.
